# The usefulness of combining narrow-band imaging with magnifying endoscopy and 18F-fluorodeoxyglucose positron emission tomography for predicting the depth of invasion in superficial esophageal squamous cell carcinoma

**DOI:** 10.1007/s10388-025-01118-7

**Published:** 2025-03-21

**Authors:** So Kodama, Kenta Watanabe, Tamotsu Matsuhashi, Sho Fukuda, Yosuke Shimodaira, Yushi Nagaki, Akiyuki Wakita, Yusuke Sato, Tomoki Tozawa, Yuki Wada, Naoko Mori, Hiroshi Nanjo, Katsunori Iijima

**Affiliations:** 1 Department of Gastroenterology, Akita University Graduate School of Medicine, 1-1-1 Hondo, Akita, 010-8543 Japan; 2 Department of Thoracic Surgery, Akita University Graduate School of Medicine, Akita, Japan; 3 Department of Radiology, Akita University Graduate School of Medicine, Akita, Japan; 4 Department of Clinical Pathology, Akita University Hospital, Akita, Japan

**Keywords:** Esophageal squamous cell carcinoma, Narrow-band imaging, 18F-fluorodeoxyglucose positron emission tomography

## Abstract

**Background:**

To ascertain the indication of endoscopic resection (ER) for esophageal squamous cell carcinoma (ESCC), accurate preoperative diagnosis of the tumor depth beyond cT1b-SM2 is crucial. This study aimed to assess the efficacy of the combined approach utilizing narrow-band imaging with magnifying endoscopy (NBI-ME) and 18F-fluorodeoxyglucose positron emission tomography (FDG-PET) for accurate discrimination of lesions of pT1b-SM2 or deeper.

**Methods:**

Between 2016 and 2023, we retrospectively enrolled 127 cases (137 lesions) of superficial, treatment-naïve ESCC at Akita University Hospital, involving patients who underwent either ER alone or surgery alone. All patients underwent preoperative NBI-ME and FDG-PET. Preoperative tumor depth was estimated using type B vessels based on NBI-ME and SUVmax based on FDG-PET, and we confirmed the final tumor depth through histopathological evaluation of resected samples. The diagnostic performance of the tests in discriminating pT1b-SM2 or deeper was evaluated in terms of sensitivity, specificity, and accuracy.

**Results:**

Treatment consisted of ER in 97 lesions and surgery in the remaining 40. Fifty-three lesions (44.7%) had pT1b-SM2 or deeper invasion. The sensitivity, specificity, and accuracy of NBI-ME using type B3 vessels were 41.5%, 97.6%, and 75.9%, respectively. For FDG-PET with a cutoff SUVmax of ≥ 2.4, these were 79.2%, 69.0%, and 73.0%, respectively. Combining both tests substantially improved diagnostic performance, with sensitivity, specificity, and accuracy of 83.0%, 89.3%, and 86.9%, respectively.

**Conclusion:**

The combination of FDG-PET and NBI-ME offers enhanced diagnostic performance for ESCC with ≥ pT1b-SM2, thereby facilitating a more efficacious preoperative narrowing of the indications for ER of superficial ESCC.

**Supplementary Information:**

The online version contains supplementary material available at 10.1007/s10388-025-01118-7.

## Introduction

Esophageal squamous cell carcinoma (ESCC) is the most common histological type of esophageal cancer in Japan, accounting for 80–90% of esophageal cancers. In the case of superficial ESCC, endoscopic resection (ER), which is minimally invasive to the patient, is feasible [1–3]. Following the 2022 edition of the Japanese Esophageal Society guidelines for treating esophageal cancer, tumor invasion up to the T1a-lamina propria mucosae (LPM) represents an absolute indication for ER, irrespective of the tumor size or location. Further, invasion of the T1a-muscularis mucosae (MM) or upper T1b-submucosa (SM) 1 represents a relative indication for ER [4]. However, patients suspected of cT1b-SM2 or deeper invasion on preoperative evaluation are candidates for surgical intervention or chemoradiotherapy. Consequently, if a pathological diagnosis of the endoscopically resected specimen ultimately confirms the pT1b-SM2 or deeper invasion of ESCC, additional treatments, such as surgery or chemoradiotherapy, are essential to achieve a cure. However, given the physical and psychological burden on the patient and the time loss associated with ER, it is preferable to diagnose cTlb-SM2 or deeper disease with greater accuracy before initiating treatment. Therefore, it is imperative to identify cT1b-SM2 or deeper invasive lesions unsuitable for ER.

Currently, using narrow-band imaging with magnifying endoscopy (NBI-ME), intratumoral microvessels (type B vessels) can be classified into types B1, B2, and B3 according to the Japan Esophageal Society (JES) classification, allowing the estimated depth of tumor invasion to be determined [5]. However, the diagnostic performance of type B vessels using NBI-ME is known to be insufficient, especially for type B2 [6–8]. Furthermore, a recent multicenter study in Japan demonstrated that endoscopic ultrasonography did not yield additional improvement in differentiating cT1b-SM2 or deeper invasive lesions compared with NBI-ME alone [9].

^18^F-fluorodeoxyglucose positron emission tomography (FDG-PET), widely used for ESCC, can visualize metabolic activity within tumor cells [10–19]. In addition, the maximum standard uptake value (SUVmax) in FDG-PET has been reported to correlate with the depth of ESCC and is considered an expected aid in diagnosing depth [20–23]. Nevertheless, few studies have investigated the most crucial diagnosis using FDG-PET, whether the tumor invasion depth is cT1b-SM2 or more, regarding the indication for ER in ESCC.

Further, to date, only a single study attempted to combine NBI-ME and FDG-PET to assess the depth of ESCC [24]. In this study, we aimed to determine whether the combination of NBI-ME and FDG-PET is effective in identifying lesions of pT1b-SM2 or deeper among the entire spectrum of superficial ESCCs.

## Material and methods

### Patients

At Akita University Hospital (Akita, Japan), between January 2016 and January 2023, patients with no history of upper gastrointestinal surgery who underwent preoperative FDG-PET and endoscopic observation, including NBI-ME, and were histologically diagnosed with esophageal ESCC were initially selected. Subsequently, individuals who had undergone preoperative chemotherapy or chemoradiotherapy, as well as those diagnosed with invasive cancer extending beyond the pT2-muscularis propria (MP) based on postoperative histological findings, were excluded from the study.

### FDG-PET protocol and assessment

The scan was performed at least two weeks before treatment using a discovery ST Elite (General Electric Company, United States of America), combined FDG-PET and CT scanner. Patients fasted for at least 6 h and had the blood glucose level controlled to less than 140 mg/dL before FDG administration.FDG-PET revealed corresponding SUVmax for primary lesions. SUV was defined as follows: SUV = radioactive concentration of tissue or lesion (MBq/mL)/injection dose (MBq)/patient weight (g).

### Endoscopic evaluation

Before treatment, the invasion depth of all lesions was estimated according to the JES classification using a magnifying endoscope (GIF-H260Z, GIF-H290Z, or GIF-XZ1200; Olympus Corporation, Tokyo, Japan). Under NBI-ME, the depth of the lesions was estimated according to the presence of the microvessels within the lesions (type B vessels, types B1, B2, and B3), with type B1 considered cT1a-epithelium (EP)/cT1a-LPM, type B2 cT1a-MM/cT1b-SM1, and type B3 cT1b-SM2 or deeper. Two endoscopists (SK and KW) independently and blindly assessed the depth of invasion of all lesions endoscopically using still images according to the JES classification [25]. In cases where their opinions differed, a joint review was conducted to reach a consensus. The macroscopic type of the lesions was classified according to the Japanese Classification of Esophageal Cancer: types 0-IIa and 0-Is as elevated, and all other types non-elevated. Tumor circumference was expressed as a quadrant and classified according to the following criteria: < 1/4, ≥ 1/4 –  < 1/2, ≥ 1/2 – < 3/4, ≥ 3/4 –  < 1, and all around.

### Histopathological assessment

After ER, resected specimens were sectioned at 2–3 mm intervals to assess histopathological tumor depth. The surgical cases were sectioned at 5 mm intervals. According to the esophageal treatment guidelines, tumors were classified for invasion depth as follows: pT1a-EP, cancer confined to the epithelium; pT1a-LPM, cancer confined to the lamina propria; pT1a-MM, cancer invading the muscularis mucosa; pT1b-SM1: cancer confined to the submucosa within 200 μm from the muscularis mucosa; and pT1b-SM2: cancer invading the submucosa more than 200 μm from the muscularis mucosa. The long axis diameter of the cancerous area in the pathological specimen was considered tumor diameter. In this study, pathologists assessed the invasion depth of all lesions.

### Statistical analyses

Continuous variables were expressed as median and interquartile range and compared using the Mann–Whitney U test. Categorical variables were expressed as numbers and percentages and compared using Fisher’s exact test. We evaluated endoscopic and FDG-PET examinations concerning sensitivity, specificity, positive predictive value (PPV), negative predictive value (NPV), and accuracy. The c-statistic was applied to evaluate the discriminative ability of PDG-PET-based SUVmax for pT1b-SM2 or deeper tumor invasion. The cutoff value for discriminating deep invasion was determined using Youden’s index. To identify associated factors regarding pT1b-SM2 or deeper tumor invasion, odds ratios (ORs) and 95% confidence intervals (CIs) were calculated using logistic regression analysis. All analyses were conducted using EZR version 1.63 (Saitama Medical Center, Jichi Medical University, Saitama, Japan) [25], and considered statistically significant as *p* < 0.05.

## Result

### Factors associated with histopathological depth

Of the 525 selected superficial ESCC cases, excluded cases were 377 who had received preoperative chemotherapy or chemoradiotherapy and 21 who had been diagnosed with pT2-MP or deeper invasive cancer. A total of 127 cases, including 8 cases with two lesions and 1 case with three lesions, fulfilled the defined criteria, totaling 137 ESCC lesions were analyzed in this study (Supplementary Fig. 1). Ninety-seven lesions (70.8%) were treated with ER and the remaining 40 (29.2%) with surgery. Fifty-three lesions (44.7%) had reached pT1b-SM2 or deeper invasive lesions. When comparing tumor depth between pT1a-EP–pT1b-SM1 and pT1b-SM2/SM3, the former had fewer elevated lesions than the latter (16.7% vs. 67.9%, P < 0.001) (Supplementary Table 1).

### Diagnostic performance of type B vessels

Table 1 shows the results of tumor depth assessment by NBI-ME. Fourty-nine of 56 type B1 lesions (87.5%) and 22 of 24 type B3 lesions (91.7%) were consistent with their corresponding histopathological types, pT1a-EP/LPM and pT1b-SM2/SM3, respectively. However, only 19 of 57 type B2 lesions (33.3%) were consistent with the corresponding histopathological type, pT1a-MM/pT1b-SM1. The specificity and PPV of type B1 for pT1a-EP/LPM and type B3 for pT1b-SM2/SM3 were relatively high, with type B1 having exceptionally high diagnostic performance (e.g., accuracy of 89.1). On the other hand, the diagnostic performance, including specificity and PPV, of type B2 was lower than that of types B1 and B3, and more than half (52.6%) of type B2 lesions estimated to be pT1a-MM/pT1b-SM1 were pT1b-SM2/SM3.

**Table 1:** Relationship between JES classification and histopathological depth

JES classification	Histopathological depth			Diagnostic performance				
	pT1a-EP/LPM	pT1a-MM/T1b-SM1	pT1b-SM2/SM3	Sensitivity, %	Specificity, %	PPV, %	NPV, %	Accuracy, %
Type B1 (cT1a-EP/LPM), *n* (%)	49 (86.0%)	6 (22.2%)	1 (1.9%)	86.0	91.2	87.5	90.1	89.1
Type B2 (cT1a-MM/T1b-SM1), *n* (%)	8 (14.0%)	19 (70.4%)	30 (56.6%)	70.4	65.5	33.3	90.0	66.4
Type B3 (cT1b-SM2/SM3), *n* (%)	0 (0.0%)	2 (7.4%)	22 (41.5%)	41.5	97.6	91.7	72.6	75.9

*EP* epithelium, *JES* the Japan Esophageal Society, *LPM* lamina propria mucosae, *MM* mucularis mucosae, *NPV* negative predictive value, *PPV* positive predictive value, *SM* submucosa

### Diagnostic performance of FDG-PET in discriminating pT1b-SM2 or deeper invasive lesions

**Fig. 1:**
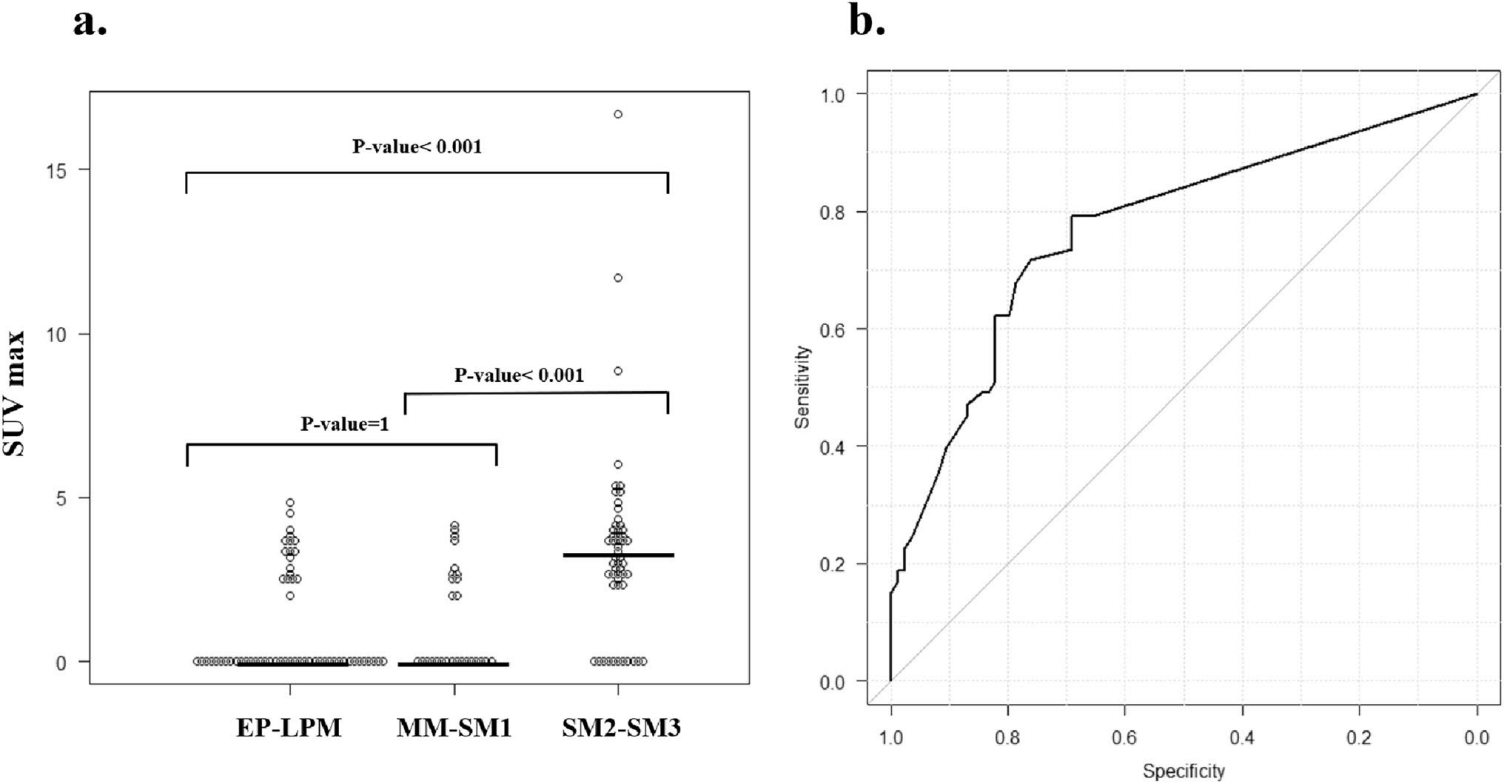
**A** Relationship between SUVmax and histopathological depth of ESCC. The median of SUVmax corresponding to pT1a-EP-LPM, pT1a-MM/pT1b-SM1, and pT1b-SM2/SM3 was 0, 0, and 3.2, respectively. **B** Receiver operating characteristic curves for discriminating pT1b-SM2 or deeper invasive lesions according to SUVmax. The c-statistic (95% CI) was 0.77 (0.69–0.85), and an SUVmax of 2.4 was considered the ideal cutoff. *CI* confidence interval, *EP* epithelium, *ESCC* esophageal squamous cell carcinoma, *LPM* lamina propria mucosae, *MM* muscularis mucosae, *SM* submucosa, *SUV* standard uptake value

Figure 1a shows the distribution of SUVmax according to 3 groups by the tumor depth. SUVmax completely overlapped between pT1a-EP/LPM and pT1a-MM/pT1b-SM1 lesions, and the majority (83.3%) of both groups showing undetectable FDG uptake, with a median value of 0. In contrast, the majority of pT1b-SM2/SM3 lesions had detectable FDG uptake, with a median SUVmax of 3.2, significantly higher than the combined group of pT1a-EP–pTb-SM1 lesions (P < 0.001), suggesting the potential value of FDG-PET in discriminating pT1b-SM2/SM3 lesions from pT1a-EP–pTb-SM1 lesions.

Subsequently, the c-statistic (95% CI) of SUVmax for discriminating pT1b-SM2/SM3 lesions was 0.77 (0.69–0.85), with an SUVmax of 2.4 as the optimal cutoff value based on Youden’s index (Fig. 1b). Under this cutoff, 42 of 53 pT1b-SM2/SM3 lesions (79.2%) had SUVmax ≥ 2.4 (Supplementary Table 2). However, 84 pT1a-EP–pTb-SM1 lesions (31.0%) had anSUVmax ≥ 2.4. Thus, FDG-PET showed relatively high sensitivity (79.2%) and NPV (84.4%) while showing relatively low specificity (69.0%) and PPV (61.8%) (Table 3).

### Development of a combined diagnostic scheme of NBI-ME and FDG-PET (N-P category)

**Fig. 2:**
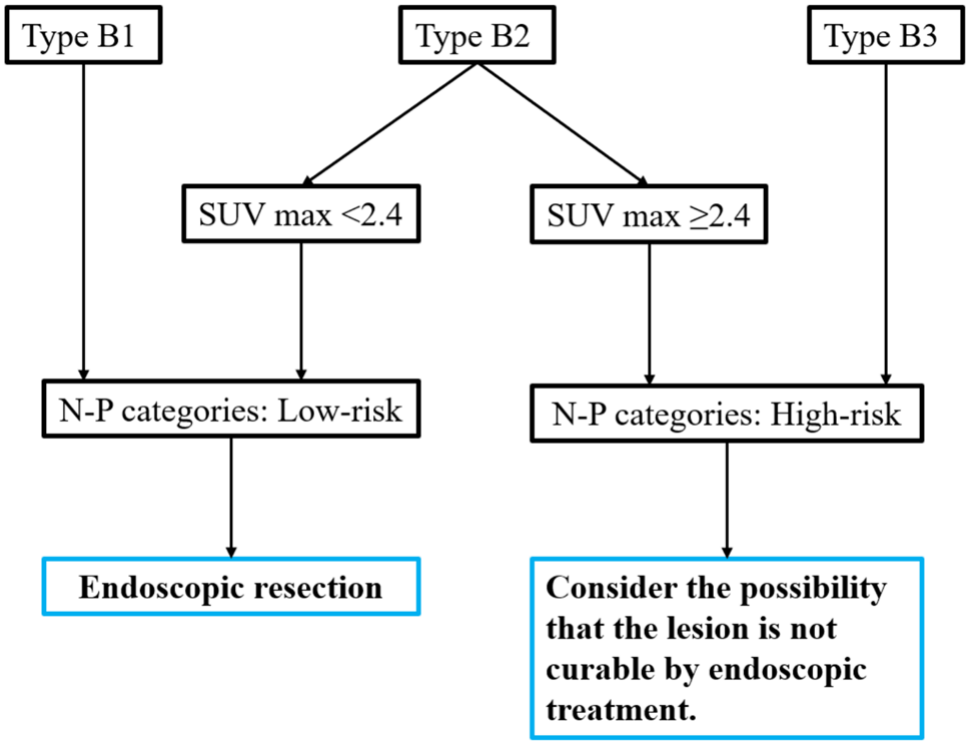
Suggested treatment strategy using N-P category. Endoscopic radical resection may be difficult for high-risk in the N-P category

While the overall accuracy of NBI-ME and FDG-PET was comparable in the current study (75.9% vs. 73.0%), revealing their details differed considerably. Regarding specificity and PPV, NBI-ME demonstrated significant advantages, especially for types B1 and B3. For instance, types B1 and B3 based on NBI-ME demonstrated specificities of 91.2% and 97.6% and PPVs of 87.5% and 91.7%, respectively (Table 1). In contrast, FDG-PET demonstrated significant advantages regarding sensitivity and NPV, with respective values of 79.2% and 84.1% (Table 3). Accordingly, we integrated these two modalities and developed a novel diagnostic scheme, N-P category, to potentially improve the discriminative performance of pT1b-SM2 or deeper ESCC lesions by utilizing their complementary characteristics (Fig. 2).

In that category, once identified as types B1 or B3 by NBI-ME, given its high specificity and PPV, diagnosis of cT1a-EP/LPM or cT1b-SM2/SM3, respectively, is permissible without the necessity of further FDG-PET examination. In contrast, regarding type B2 by NBI-ME, the diagnostic performance is relatively low, suggesting a benefit to adding FGD-PET testing with high sensitivity and NPV. In other words, lesions with type B2 plus SUVmax < 2.4 are more likely to be pT1a-EP-pTb-SM1, while those with type B2 plus SUVmax ≥ 2.4 are more likely to be pT1b-SM2/SM3. Therefore, in the N-P category for predicting pT1b-SM2/SM3 invasion, lesions of type B1 with any SUVmax or type B2 with SUVmax < 2.4 were considered low-risk, and lesions of type B2 with SUVmax ≥ 2.4 or type B3 with any SUVmax were considered high-risk (Fig. 2).

### Discrimination and diagnostic performance of N-P category for SM2 or deeper invasive lesions

Under the N-P category, 75 of 84 low-risk (89.3%) and 44 of 53 high-risk (83.0%) lesions had the corresponding histopathological types, pT1a-EP–pT1b-SM1 and pT1b-SM2/SM3, respectively (Table 2). The sensitivity, specificity, PPV, NPV, and accuracy of the N-P category for predicting pT1b-SM2/SM3 invasion were 83.0%, 89.3%, 83.0%, 89.3%, and 86.9%, respectively. It is noteworthy that the accuracy was substantially enhanced by the N-P category (86.9%) in comparison to NBI-ME alone (75.9%) or FDG-PET alone (73.0%) (Table 3).

**Table 2:** Relationship between N-P category and histopathological depth

N-P category	Histopathological depth		*p* value
	pT1a-EP–T1b-SM1 (*n* = 84)	pT1b-SM2/SM3 (*n* = 53)	
Low-risk^†^, *n* (%)	75 (89.3)	9 (17.0)	< 0.001
High-risk^‡^, *n* (%)	9 (10.7)	44 (83.0)	

*EP* epithelium, *SM* submucosa, *SUV* standardized uptake value

^†^Lesions of types B1 with any SUVmax or B2 with SUVmax < 2.4

^‡^Lesions of types B2 with SUVmax ≥ 2.4 or B3 with any SUVmax

**Table 3:** Diagnostic performances of JES classification, SUVmax of ≥ 2.4, and high-risk in the N-P category for estimating pT1b-SM2 or deeper invasive lesions

Diagnostic method	Sensitivity	Specificity	PPV	NPV	Accuracy
JES classification (type B3)	41.5	97.6	91.7	72.6	75.9
SUVmax ≥ 2.4	79.2	69.0	61.8	84.1	73.0
High-risk in N-P category^†^	83.0	89.3	83.0	89.3	86.9

*JES* the Japan Esophageal Society, *PPV* positive predictive value, *NPV* negative predictive value, *SUV* standardized uptake value

^†^Lesions of types B2 with SUVmax ≥ 2.4 or B3 with any SUVmax

Since the elevated type of ESCC is known to be associated with pT1b-SM2 or deeper invasion, we further performed regression analyses to ascertain the usefulness of the N-P category independent of morphological features (e.g., elevated or non-elevated). Multivariable regression analysis demonstrated that high-risk by the N-P category was strongly associated with pT1b-SM2 or deeper invasion regardless of morphological features with an adjusted OR (95% CI): 36.1 (11.1–118.0) (supplemental Table 3).

## Discussion

Preoperative diagnosis of T1b-SM2 or deeper invasive lesions is critical in selecting appropriate indications for ER for ESCC. This study demonstrated that combining NBI-ME and FDG-PET significantly improved the ability to discriminate pT1b-SM2 or deeper invasion compared with either procedure alone.

Estimating tumor depth according to type B vessels by NBI-ME is a widely used endoscopic diagnostic technique. Nevertheless, the diagnostic efficacy of type B vessels may be inadequate. In particular, the diagnostic accuracy of type B2, originally defined as an indicator of pT1a-MM/p-T1b-SM1, has been demonstrated to be inferior to types B1 and B3. Although Oyama et al. initially reported a very high accuracy (93.4%) for type B2-vessels [26], subsequent studies have reported lower accuracy rates (76.8%, 78.6%) [8, 24]. Indeed, the latest high-quality prospective multicenter study demonstrated that the accuracy of NBI-ME with all types of B vessels for discriminating pT1b-SM2/SM3 was relatively low (72.9%), even among the leading high-volume institutes in Japan [9]. Additionally, the issue of only a moderate level of interobserver agreement for type B2 (κ = 0.64) is noteworthy [8], as it may contribute to variability in outcomes across studies. Consistent with previous studies, the diagnostic accuracy of type B2 in the current study was 66.4%, lower than that of types B1 and B3.

Given the positive correlation between SUVmax and tumor depth, FDG-PET may effectively estimate that of ESCC. However, FDG-PET alone is insufficient to make a sensitive estimate of whether a lesion has invaded SM2 or deeper regarding the indication for ER. In fact, in this study, the accuracy of FDG-PET for predicting pT1b-SM2 or deeper invasion was relatively low (73.0%), and a significant proportion of cases showed false positives, even in lesions of shallow depths, such as pT1a-EP/LPM (see Fig. 1A).

Consequently, focusing on the high specificity and PPV in types B1 and B3 by NBI-ME and high sensitivity and NPV by FDG-PET, a new diagnostic scheme (N-P category) has been developed by combining them. In the N-P category, to predict pT1b-SM2/SM3 invasion, lesions of type B1 with any SUVmax or type B2 with SUVmax < 2.4 were considered low-risk, and lesions of type B2 with SUVmax ≥ 2.4 or type B3 with any SUVmax were considered high-risk. The result showed that the sensitivity, specificity, PPV, NPV, and accuracy of the high-risk N-P category were 83.0%, 89.3%, 83.0%, 89.3%, and 86.9%, respectively, a notable improvement in diagnostic performance when compared to the individual tests alone. It may thus not be advisable to consider high-risk lesions in the N-P category as indications for endoscopic treatment. In this study, although more than half (30/57) of lesions with clinically type B2 were pT1b-SM2/SM3 instead of the corresponding correct histopathology, pT1a-MM/pT1b-SM1, the combined assessment using SUVmax ≥ 2.4 may be beneficial in identifying additional pT1b-SM2 or deeper lesions problematic to estimate by NBI-ME alone. The new N-P category successfully reduced the number of such overlooked cases from 30 (52.6%) to 9 (17.0%) (see Tables 1 and 2).

The present state of the N-P categorization system is not without fault. For instance, the category can be used to directly select surgery or chemoradiotherapy based on N-P category in the absence of histological confirmation of ER-resected specimens, which could result in overtreatment in 9 (17.0%) cases. In consideration of the patients' substantial burden from surgery and chemoradiotherapy, the overtreatment of patients should be avoided to the greatest extent possible. Notwithstanding these limitations, the N-P category provides both physicians and patients with a more precise estimation of the likelihood of pTlb-SM2 or deeper, and it facilitates shared decision-making regarding the selection of treatment for superficial ESCC.

To date, only a single study aimed to integrate NBI-ME and FDG-PET to improve the diagnostic efficacy of ESCC with ≥ pT1b-SM2. Toriyama and colleagues demonstrated that the combined diagnostic scheme exhibited a sensitivity, specificity, PPV, NPV, and accuracy of 78.3%, 91.5%, 78.3%, 91.5%, and 87.8%, respectively, with values comparable to those observed in the present study. They concluded that the combination enhanced the diagnostic performance compared to either test alone [24]. Similar to the present study, this pioneering study employed a diagnostic scheme combining two tests, although the number of ESCC lesions was relatively small (n = 82), especially only 15 with ≥ pT1b-SM2. Therefore, the study findings may not be conclusive. In the present study, we included a lot more ESCC lesions (n = 137), especially a significantly higher number of ≥ pT1b-SM2 lesions (n = 53), allowing us to draw more robust conclusions. Furthermore, in contrast to the preceding study, determining the appropriate cutoff for FDG-PET allowed for a quantitative evaluation to diagnose tumor depth. The quantitative classification of lesions with type B2, which may exhibit various tumor depths (T1a-EP/LPM, Tla-MM/T1b-SM1, or Tb1-SM2/SM3), into two groups, low-risk and high-risk, using FDG-PET with appropriate cutoffs is a valuable approach to enhance the diagnostic performance for tumor depth.

Several limitations of this study should be noted. First, as this is a single-center retrospective study, the number of subjects is relatively small, and selection bias may have been present. In our hospital, FDG-PET was to be routinely performed before treatment to determine the stage of ESCC. However, in many type B1 lesions identified by NBI-ME, where the lesions were considered shallow, FDG-PET was omitted due to cost-effectiveness considerations (e.g., 196 lesions of type B1 were treated by ER without FDG-PET assessment during a 7-year enrollment period). Such cases were not included in the present study, potentially introducing selection bias. Nevertheless, the exclusion is expected to have a minimal impact on diagnostic performance for type B2 lesions, which is the main interest of this study. Second, variations in the optimal cutoff value for SUVmax may exist depending on the PET-CT scanner used. Lastly, this study was conducted at a single center and has not been validated. Further prospective studies in different settings are warranted.

In conclusion, preoperative evaluation with a combination of FDG-PET and NBI-ME improves diagnostic performance for ESCC with ≥ pT1b-SM2, mainly by quantitatively classifying ambiguous lesions with type B2 into two groups: high-risk and low-risk, using a cutoff of SUVmax ≥ 2.4. Combining the two tests potentially facilitates a more efficacious preoperative narrowing of the indications for ER of superficial ESCC.

## Supplementary Information

Below is the link to the electronic supplementary material.

Supplementary file1 (PDF 315 KB)

## Data Availability

Data supporting the findings of this study are available from the corresponding author upon reasonable request.
