# Supplementary material for: The usefulness of combining narrow-band imaging with magnifying endoscopy and 18F-fluorodeoxyglucose positron emission tomography for predicting the depth of invasion in superficial esophageal squamous cell carcinoma
Source: Esophagus. 2025 Mar 21;22(3):437–43. doi: 10.1007/s10388-025-01118-7 (PMC12167288; doi:10.1007/s10388-025-01118-7)
Supplement: Supplementary file 1 — Supplementary file1 (PDF 315 KB) [file 10388_2025_1118_MOESM1_ESM.pdf]

Supplementary Table 1. Clinicopathological characteristics of study patients

	Entire subjects	Histopathological depth		*p* value
		**pT1a-EP–pT1b-SM1 (*n* = 84)**	**pT1b-SM2/SM3 (*n* = 53)**	
Age (years), median (IQR), y	70.0 (63.0–75.0)	70 (63.0–76.3)	71 (62.0–75.0)	0.470
Sex, *n* (%)				
Male	126 (92.0)	76 (90.5)	50 (94.3)	0.529
Female	11 (8.0)	8 (9.5)	3 (5.7)	
Body mass index (kg/m^2), median (IQR)	21.1 (19.1-23.3)	21.6 (19.3-23.8)	20.0 (19.0-22.1)	0.073
Tumor location, *n* (%)				
Cervical esophagus	5 (3.6)	2 (2.4)	3 (5.7)	0.508
Upper thoracic esophagus	23 (16.8)	12 (14.3)	11 (20.8)	
Middle thoracic esophagus	72 (52.6)	44 (52.4)	28 (52.8)	
Lower thoracic esophagus	32 (23.4)	23 (27.4)	9 (17.0)	
Esophagogastric junctional zone	5 (3.6)	3 (3.6)	2 (3.8)	
Macroscopic type, *n* (%)				
Elevated type	50 (36.5)	14 (16.7)	36 (67.9)	<0.001
Non-elevated type	87 (63.5)	70 (83.3)	17 (32.1)	
Tumor diameter (mm), median (IQR)	28.0 (18.0-41.0)	28.0 (17.0-45.0)	28.0 (20.0-40.0)	0.889
Tumor circumference, *n* (%)				
<1/4	44 (32.1)	33 (39.3)	11 (20.8)	0.178
≥1/4 to <1/2	34 (24.8)	19 (22.6)	15 (28.3)	

≥1/2 to <3/4	31 (22.6)	15 (17.9)	16 (30.2)
≥3/4 to <1	18 (13.1)	11 (13.1)	7 (13.2)
All around	10 (7.3)	6 (7.1)	4 (7.5)

EP, epithelium; IQR, interquartile range; LPM, lamina propria mucosae; MM, muscularis mucosae; SM, submucosa.

Supplementary Table 2. Relationship between SUVmax and histopathological depth

SUVmax	Histopathological depth		*p* value
	**T1a-EP–T1b-SM1 (*n* = 84)**	**T1b-SM/SM3 (*n* = 53)**	
<2.4, n (%)	58 (84.1)	11 (15.9)	<0.001
≥2.4, n (%)	26 (38.2)	42 (61.8)	

EP, epithelium; SM, submucosa; SUV, standardized uptake value

Supplementary table 3. Factors associated with pT1b-SM2 or deeper invasive lesions

	pT1b-SM2/SM3 lesions, *n*/total (%)	Univariable			Multivariable		
		**OR**	**95% CI**	***p* value**	**Adjusted OR** †	**95% CI**	***p* value**
N-P category							
Low-risk ‡	9/53 (17.0)	Reference			Reference		
High-risk §	44/53 (83.0)	40.70	15.00-110.00	<0.001	36.10	11.10-118.00	<0.001
Macroscopic type							
Non-Elevated type	17/53 (32.1)	Reference			Reference		
elevated type	36/53 (67.9)	10.60	4.70-23.90	<0.001	4.30	1.40-13.20	0.011

CI, confidence interval; OR, odds ratio; SM, submucosa; SUV, standardized uptake value

† Adjusted by age, sex, tumor diameter, macroscopic type, and N-P category.

‡ Lesions of types B1 with any SUVmax or B2 with SUVmax <2.4.

§ Lesions of types B2 with SUVmax≥2.4 or B3 with any SUVmax.

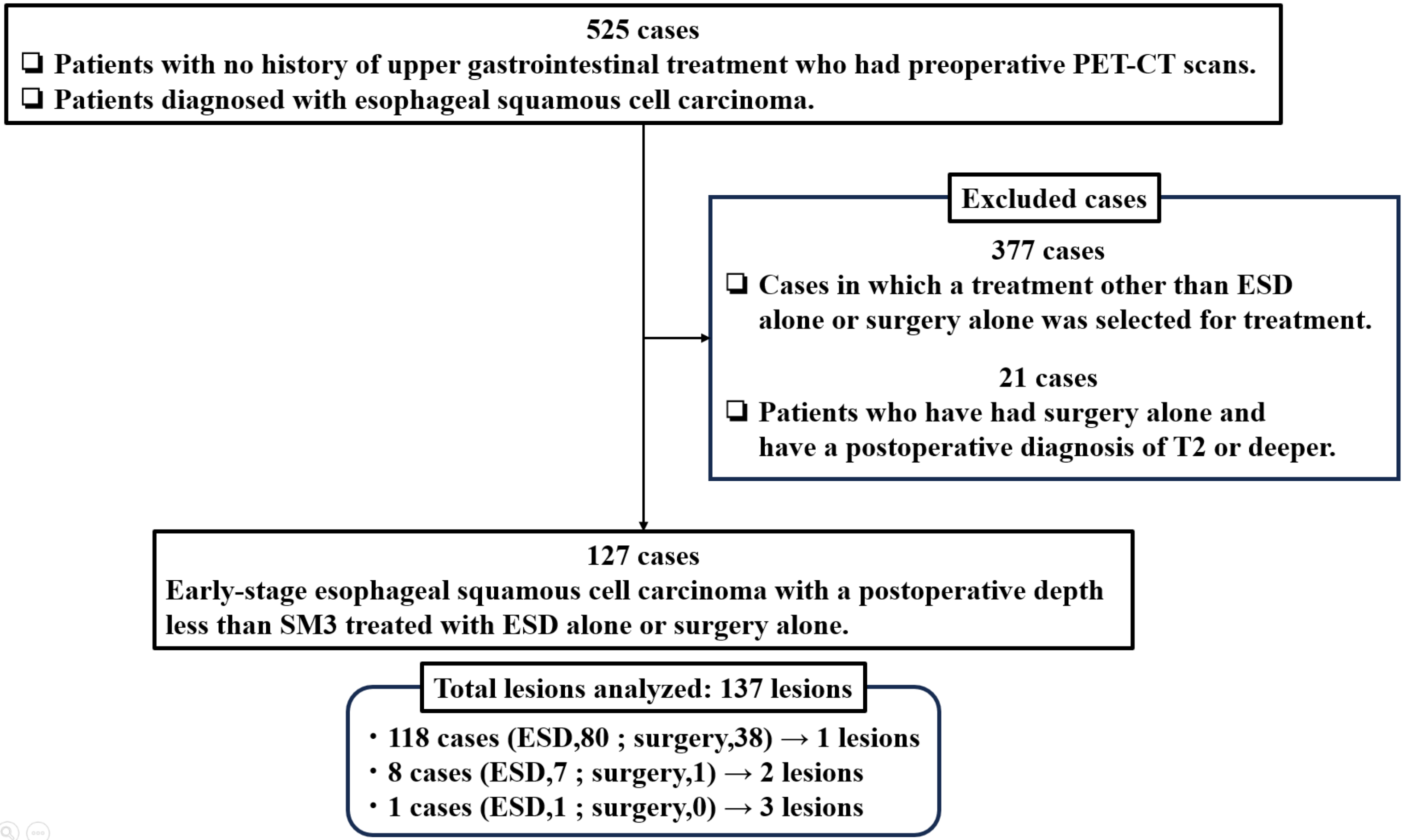

Supplementary Figure1. Flowchart for patient enrollment in this study. ER, endoscopic resection; FDG-PET, ^{18}F-fluorodeoxyglucose positron emission tomography
